# Stringent and reproducible tetracycline-regulated transgene expression by site-specific insertion at chromosomal loci with pre-characterised induction characteristics

**DOI:** 10.1186/1471-2199-8-30

**Published:** 2007-05-10

**Authors:** Rachel Brough, Antigoni M Papanastasiou, Andrew CG Porter

**Affiliations:** 1 Gene Targeting Group, Department of Haematology and MRC Clinical Sciences Centre, Faculty of Medicine, Imperial College, London, W12 0NN, UK; 2 The Breakthrough Breast Cancer Research Centre, Institute of Cancer Research, 237 Fulham Road, London, SW3 6JB, UK; 3 Institute of Child Health, 30 Guilford Street, London, WC1N 1EH, UK

## Abstract

**Background:**

The ability to regulate transgene expression has many applications, mostly concerning the analysis of gene function. Desirable induction characteristics, such as low un-induced expression, high induced expression and limited cellular heterogeneity, can be seriously impaired by chromosomal position effects at the site of transgene integration. Many clones may therefore need to be screened before one with optimal induction characteristics is identified. Furthermore, such screens must be repeated for each new transgene investigated, and comparisons between clones with different transgenes is complicated by their different integration sites.

**Results:**

To circumvent these problems we have developed a "screen and insert" strategy in which clones carrying a transgene for a fluorescent reporter are first screened for those with optimal induction characteristics. Site-specific recombination (SSR) is then be used repeatedly to insert any new transgene at the reporter transgene locus of such clones so that optimal induction characteristics are conferred upon it. Here we have tested in a human fibrosarcoma cell line (HT1080) two of many possible implementations of this approach. Clones (e.g. Rht14-10) in which a GFP reporter gene is very stringently regulated by the tetracycline (tet) transactivator (tTA) protein were first identified flow-cytometrically. Transgenes encoding luciferase, I-*Sce*I endonuclease or Rad52 were then inserted by SSR at a *LoxP *site adjacent to the GFP gene resulting stringent tet-regulated transgene expression. In clone Rht14-10, increases in expression from essentially background levels (+tet) to more than 10^4^-fold above background (-tet) were reproducibly detected after Cre-mediated insertion of either the luciferase or the I-*Sce*I transgenes.

**Conclusion:**

Although previous methods have made use of SSR to integrate transgenes at defined sites, none has effectively combined this with a pre-selection step to identify integration sites that support optimal regulatory characteristics. Rht14-10 and similar HT1080-derived clones can now be used in conjunction with a convenient delivery vector (pIN2-neoMCS), in a simple 3-step protocol leading to stringent and reproducible transgene regulation. This approach will be particularly useful for transgenes whose products are very active at low concentrations and/or for comparisons of multiple related transgenes.

## Background

The introduction of gene expression vectors into vertebrate cells is a powerful way to investigate gene function. Following transgene integration into the genome of a recipient cell, stable, heritable phenotypes are studied in cultured cells, or (if host cells are totipotent) in a developing/adult animal. Transgenes are typically used either to express or down-regulate a specific target gene, the latter being achieved via the use of RNA interference (RNAi) or site-specific recombinases (e.g. Cre) whose recognition sequences (e.g. *loxP*) have been engineered into the target gene. In cases where the gain or loss of gene function places host cells at a selective disadvantage, is lethal, or has different consequences at different developmental stages, temporally and/or spatially regulated transgene expression is necessary [1-4].

Temporally regulated transgene expression can be achieved with a variety of regulatory systems, but the most widely used are based on the tetracycline (tet) repressor TetR. The original Tet-Off system [5] is based on the tet-transactivator (tTA), a fusion protein between TetR and a minimal transactivation domain of the viral VP16 protein. In the absence of tet, tTA binds to *tetO *recognition sequences in the Tet Response Element (TRE) upstream of the gene to be regulated, and activates RNA polymerase II-dependent transcription from an adjacent minimal promoter (CMVmin). When tet is present, tTA dissociates from the TRE and transcription ceases. Many variations on the Tet-Off system have been developed (e.g. see [6-8]) often with the aim of improving stringency. A stringent system here refers to one with minimal transgene expression before induction and robust expression after induction.

Use of tet-regulated transgenes either to express or silence a target gene is already widespread in both cell lines and animals, but new applications continue to be developed. For example, with the growing use of site-specific recombinases [9,10], RNA polymerase II-based methods for stably expressing short hairpin RNA (shRNA [11,12]), and the expression of inducible transgenes in host cells with disrupted target alleles [13,14], the scope for tet-regulated gene silencing is still expanding. While good stringencies are often described in such studies, these can be time-consuming to generate and, depending on the purpose of the study, may be less than ideal.

Chromosomal position effects [15] at the site of transgene integration are key variables in the success of these approaches as they can adversely affect the stringency of tet-regulation. As a direct result, position effects increase the effort required to identify cell or animal lines with sufficiently stringent transgene regulation, and complicate the comparisons between different lines. When evaluating lines expressing two related inducible transgenes, for example, any phenotypic variations can reflect differences either between the protein products of the transgenes or, more trivially, between their induced expression levels, as determined by the transgene integration sites. Dissimilar phenotypes can also indicate dysregulation of different host genes close to the transgene integration sites [16].

To date, the influence of position effects on tet-regulation has been limited in two different ways. One method uses insulator sequences [17] to protect the transgene from surrounding sequences (and *vice versa*). While this has been useful in increasing stringency [18], variation between different clones remains. In another approach, site-specific recombination (SSR) is used to integrate tet-regulated transgenes at specific chromosomal loci [19-22]. This approach effectively eliminates variations between clones, allowing for reproducible induction characteristics, but its success in improving stringency depends on the chosen integration site and this has not been systematically optimised.

Here we describe a new approach in which clones with optimal integration sites are first identified by flow cytometric screens of cells that have been stably transfected with a *loxP*-tagged reporter transgene (encoding enhanced green fluorescent protein [EGFP]). Cre-mediated recombination is then used to insert a promoterless gene of interest (GOI) at the *loxP *site of such clones. We have validated this approach, which we term "Screen and Insert' (or ScIn), using luciferase as a test GOI and two different implementation strategies. We have also used the method to generate clones that stringently regulate the expression of I-*Sce*I, an endonuclease widely used to study cellular responses to the formation of DNA double strand breaks (DSBs) [23,24], and Rad52, a protein involved in the repair of DSBs by homologous recombination [25].

## Results

### Screen and insert: principles and implementation strategies

**Figure 1:**
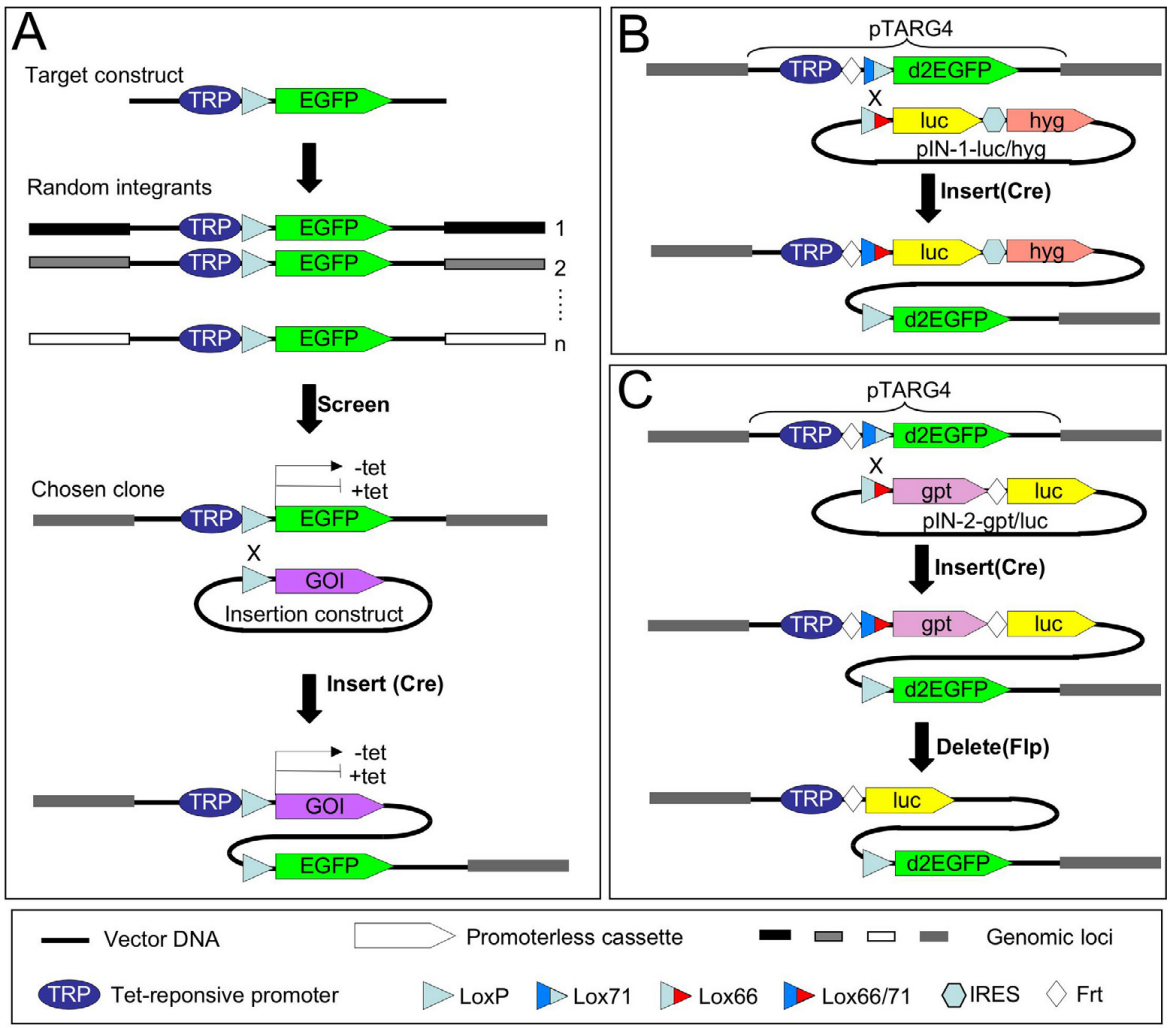
**Screen and Insert principles and strategies**. Configurations of plasmid DNA before and after its incorporation into genomic DNA are shown schematically (not to scale). A) Principles of the Screen and Insert approach. B) The Screen and Insert type 1 (ScIn-1) strategy. C) The Screen and Insert type 2 (ScIn-2) strategy. Promoterless cassettes are shown for a generic gene of interest (GOI), and for genes encoding green fluorescent proteins (EGFP and d2EGFP), luciferase (luc), hygromycin phophotransferase (hygro) and guanosine phospribosyltranferase (gpt). See text for details.

An outline of the basic Screen and Insert approach is shown in Fig. 1A. A target construct is made in which a reporter gene (*EGFP*) is expressed from a tet-responsive promoter (TRP = TRE + CMVmin) with a *loxP *site positioned between the TRP and the reporter gene. Stably transfected clones, each with the target construct randomly integrated at a different site, are screened by flow cytometry to identify those with stringently tet-regulated EGFP expression. A chosen clone is then co-transfected with a Cre-expression plasmid and an insertion construct in which a promoterless GOI cassette is linked to a *loxP *site. Cre-mediated recombination between the insertion construct and the integrated target construct places the GOI under the control of the TRP in the chosen clone. Correct insertion events generate clones with stringently tet-regulated GOI and no GFP expression.

In the present study we have tested two of many possible ways in which the basic ScIn strategy can be implemented. We will refer to these as ScIn-1 and ScIn-2 and their key features are summarised in Fig. 1B and 1C, respectively. The target construct (pTARG4) used for both ScIn-1 and ScIn-2 carries a destabilised EGFP reporter gene (d2EGFP) driven by an optimised TRP (from pTRE-tight, Clontech). Positioned between the TRP and d2EGFP are a recognition site (FRT) for the Flp site-specific recombinase, and a mutant *loxP *site, *lox71*. The FRT site is necessary for ScIn-2 only. *Lox71 *was chosen because it is known to undergo unidirectional Cre-dependent recombination with *lox66 *[26]. Thus, by use of a *lox66 *site in the insertion construct, unimolecular Cre-mediated excision events that reverse the desired integration events, are minimised.

In ScIn-1, the GOI (e.g. luciferase) is linked to a promoterless drug resistance marker gene (e.g. *hyg*) by an internal ribosome entry site (IRES). With this arrangement it is possible to select in drug (hygromycin) for the desired insertion events. A limitation of ScIn-1 is that for drug selection to work the GOI must also be expressed. In ScIn-2 this limitation is avoided by use of an insertion construct in which a promoterless drug-resistance gene (e.g. *gpt*) is linked to the GOI (luciferase) by an FRT site. This arrangement allows insertion events to be selected in drug (Mycophenolic acid and xanthine [MPA/X]) without any accompanying GOI expression. Efficient Flp-mediated excision is then used to delete the drug-resistance marker and bring the GOI under the control of the TRP, in the presence of tet.

### Screening: identification of clones with stringently regulated target constructs

**Figure 2:**
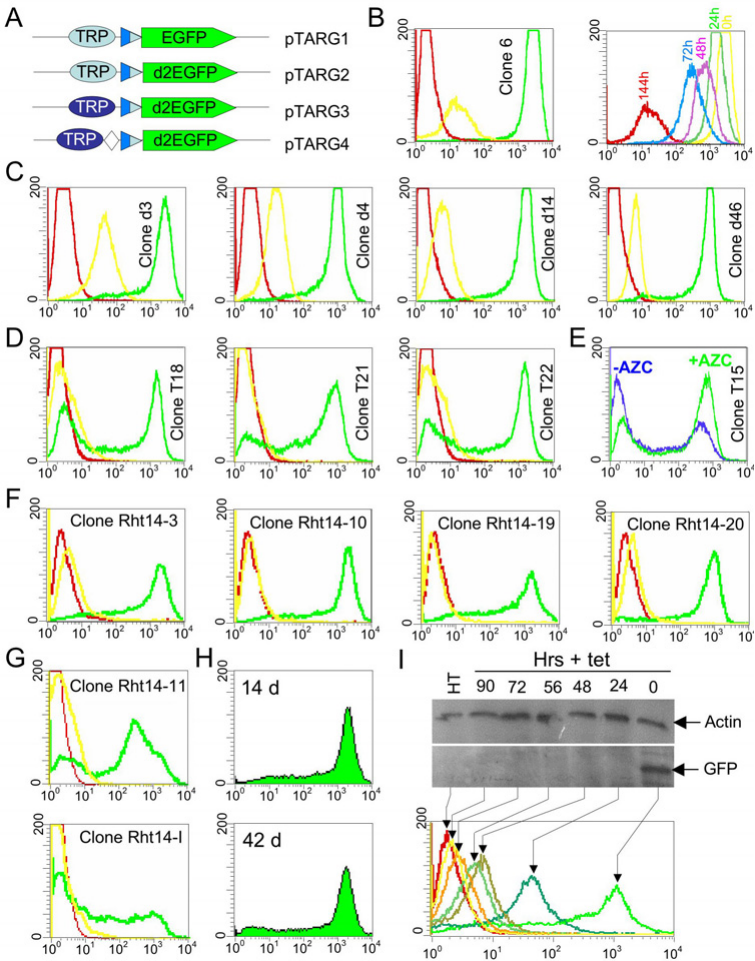
**Screening for well-regulated GFP expression**. **A**. Schematic (not to scale) of the target constructs used. DNA is represented as in Fig. 1. Pale and dark ellipses represent original and improved TRP, respectively. **B-I**. Flow cytometric profiles (y-axis, counts; x-axis, GFP fluorescence [FLH1]) of clones isolated after transfection with plasmids shown in A. For profiles in B (left only), C, D, F and G the green and yellow traces represent cells grown with or without tet, respectively, and the red traces represent GFP-negative cells (Rht14 or HT1080). **B**. Left: Clone 6, the most stringently regulated clone isolated after transfection of pTARG1 into HT2 cells. Right: GFP expression profile of Clone 6 at indicated times after addition of tet. **C**. Four of the most stringently regulated clones isolated after transfection of pTARG2 into HT2 cells. **D**. Three of the most stringently regulated clones isolated after transfection of pTARG3 into HT2 cells. **E**. Effect of AZC on GFP expression in T15, a pTARG3-transfected HT2 clone. Cells were cultured with (green) or without (blue) AZC for 24 h, and then for a further 24 h without AZC, before analysis. **F**. Four of the most stringently regulated clones isolated after transfection of pTARG4 into Rht14 cells. **G**. GFP expression profile of two heterogeneously expressing clones isolated after transfection of Rht14 with pTARG4. **H**. Profile of clone Rht14-10 grown continuously with passaging for 14 d or 42 d. **I**. GFP expression in clone Rht14-19, as measured by immunoblot (top) or flow cytometry (bottom), at the indicated times after addition of tet.

Target constructs (Fig. 2A) were introduced into host cells by co-electroporation with a plasmid (pBLpuroR) conferring resistance to Puromycin (see methods). Two HT1080 (human fibrosarcoma) derivatives were used as host cells: HT2 [27], which expresses the original tTA protein, or Rht14 (see Methods) which expresses itTA [28], an improved tTA whose gene is modified to eliminate CpG dinucleotides and potential splice sites from the prokaryotic coding DNA and to introduce eukaryotic codon-usage. HT2 cells were used as a recipient for all target constructs except pTARG4, for which Rht14 was used. Most (>70%) of puromycin-resistant (puro^r^) colonies expressed GFP as judged by fluorescence microscopy. These were expanded and tested flow cytometrically for tet-regulated GFP expression (methods). We sought to identify clones with: i) high GFP expression in the absence of tet, ii) low GFP in the presence of tet (as close as possible to the background fluorescence measured in untransfected parental cells), and iii) 'single peak' profiles, i.e. uniform expression throughout the population, whether in the presence or the absence of tet.

An initial target construct (pTARG1) used the original TRE [29]. Of over 100 puro^r ^pTARG1 transfectants, many showed good regulation, but even the best expressed significant uninduced levels of GFP and required 144 h for full down-regulation (e.g. clone 6, Fig. 2B). To facilitate screening we used a second target construct (pTARG2) with d2EGFP as reporter. This reporter has reduced half-life compared to EGFP. Over 200 puro^r ^pTARG2 transfectants were screened, but none completely silenced GFP expression in the presence of tet. Flow cytometric profiles of the best-regulated clones are shown in Fig. 2C. When a target construct (pTARG3) with an improved TRE was used, 8 of 63 puro^r ^clones analysed had uninduced GFP expression levels of less than twice the background level, but these showed heterogeneous GFP expression in the absence of tet (Fig. 2D). Experiments with the methylation inhibitor 5-aza-2'deoxycytidine (AZC) indicated that heterogeneous expression was caused by DNA methylation of the tTA gene [30]. For example, when T15, one of the clones transfected with pTARG3, was treated with AZC for 24 h, a marked increase in GFP-expressing cells was observed (Fig. 2E). Furthermore, luciferase expression in the same clone transiently transfected with a luciferase reporter gene linked to the improved TRE (pTIGHT-luc, Clontech) was also stimulated (2.6-fold) by a 24 h treatment of the cells with AZC prior to the transfection (Methods). This latter experiment suggested that methylation of the tTA gene, not the TRE, was responsible heterogeneous expression. The relatively homogeneous GFP expression seen in pTARG1 and pTARG2-transfected clones (Fig. 2C,D) probably reflects the use of lower passages of HT2 cells that were less likely to have suffered tTA gene silencing.

To minimise tTA gene methylation, the itTA-expressing HT1080 line Rht14 was made (Methods) and used for a final screening experiment. To allow subsequent testing of both ScIn-1 and ScIn-2, an FRT-tagged target construct (pTARG4) was used. Of 32 puro^r ^clones analysed, 7 had uninduced GFP expression levels of <2-fold above background combined with acceptable single-peak profiles (Fig. 2F), while others showed heterogeneous expression (Fig. 2G). The two best clones (Rht14-10 and Rht14-19) were chosen for further analysis.

Both Rht14-19 and Rht14-10 were found by Southern blot analysis (not shown) to carry a single copy of pTARG4. To test for stability of expression, clone Rht14-10 was cultured continuously for 42 days during which time the profile of GFP expression remained largely unchanged (Fig. 2H). A comparison of flow cytometric and immunoblot analyses of tet-induced GFP down-regulation in clone Rht14-19 illustrates one of the advantages of screening by flow cytometry, namely its sensitivity (Fig. 2I). Thus, substantial above-background GFP expression, readily detectable by flow cytometry, is undetectable on immunoblots. A second advantage of flow cytometric screening is its ability to detect and eleminate heterogeneously expressing clones, such as those in Fig. 2D and 2G, which would have been difficult or impossible to identify by bulk measurements of GFP expression (e.g. by immunoblot).

### ScIn-1 insertion

**Figure 3:**
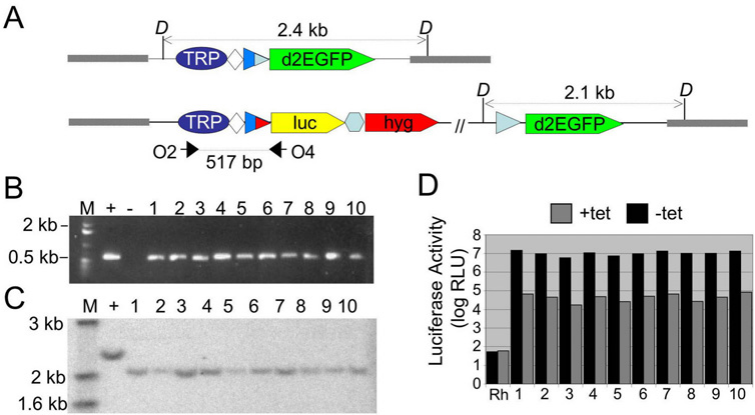
**Cre-mediated insertion by ScIn-1**. **A**. Schematic (not to scale) of the target locus in clone Rht14-19 before (top) and after (bottom) Cre-mediated insertion of pIN-1. DNA is represented as in Fig. 1 with recognition sites for *Dra*I (*D*) and PCR primers indicated. **B**. Ethidium bromide stained agarose gel of electrophoresed PCR products generated with the primers in A, and pTIGHTluc (Clontech) DNA (+), no DNA (-) or cell pellets of 10 hyg^r^, GFP-negative clones (1–10) selected after transfection of Rht14-19 with pIN-1 and pMC-Cre. M= marker DNA. **C**. Southern blots of genomic DNA isolated from Rht14-19 (+) and the same Rht14-19 derivatives described in B digested with *Dra*I. The probe was from the d2EGFP cassette. M = marker DNA. **D**. Luciferase activity in lysates from Rht14-19 (Rh) or the same Rht14-19 derivatives (1–10) described in C, grown in the absence or the presence (for 48 h.) of tet.

The insertion construct pIN-1 (Fig. 1B) was co-lipofected with a Cre expression construct (pMC-Cre) into 5 × 10^5 ^Rht14-19 cells to generate 53 hygromycin-resistant (hygro^r^) colonies. In a control experiment, identical except for the omission of pMC-Cre, no hygro^r ^colonies were generated. Of 48 hygro^r ^colonies examined, 47 had lost GFP expression and became sensitive to hygromycin when tet was added (not shown), both expected consequences of the desired integration event. DNA fragments diagnostic for the expected insertion event were detected in all (10/10) of the GFP-negative, hygro^r ^clones analysed by PCR and Southern blots (Fig. 3A–C). The same 10 clones also showed similar patterns of tet-regulated luciferase expression (Fig. 3D). These results show that the desired Cre-mediated insertion step occurs at a frequency of approximately10^-4 ^per transfected cell (Table 1) to generate tet-regulated GOI expression in a reproducible manner. Given the stringency of GFP expression in clone Rht14-19, the background of luciferase expression in the insertion clones was surprisingly high. Based on the better stringencies obtained with clone Rht14-19 using ScIn-2 (next section), this background may indicate some activation of the TRP by unidentified sequences in pIN-1.

### ScIn-2 insertion

**Figure 4:**
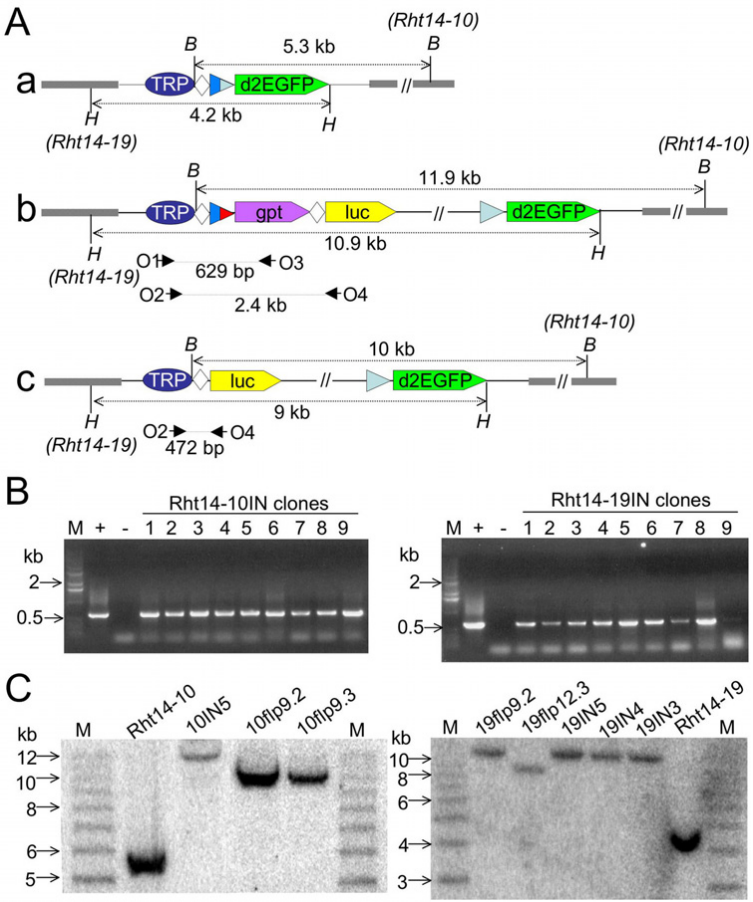
**Cre-mediated insertion by ScIn-2**. **A**. Schematic (not to scale) of the target locus in clone Rht14-19 or Rht14-10 before (a) and after (b) Cre-mediated insertion of pIN-2, and after flp-mediate deletion (c). DNA is represented as in Fig. 1 with recognition sites for *Hind*III (*H*) and *Bgl*II (*B*) and PCR primers indicated. **B**. Ethidium bromide stained agarose gel of electrophoresed PCR products generated with the primers O1 and O3 and cell pellets of 9 MPA/X^r^, GFP-negative clones selected after transfection of Rht14-10 (Rht14-10IN) or of Rht14-19 (Rht14-19IN) with pIN-2 and pMC-Cre. M = marker DNA. Negative (-) and positive (+) controls used, respectively, no DNA and pTIGHTgpt DNA (expected product: 555 bp). **C**. Southern blots of genomic DNA isolated from Rht14-10 and derivatives and digested with *Bgl*II (left), or from Rht14-19 and derivatives and digested with *Hind*III (right). Derivatives before (10IN5 and 19IN3, 4 and 5) and after (10flp9.2 and 3 and 19flp9.2 and 12.3) Flp-mediated deletion are analysed. The probe was a fragment of the *d2EGFP *gene (methods).

The insertion construct pIN-2 (Fig. 1C) was co-lipofected with pMC-Cre into 5 × 10^5 ^Rht14-10 or Rht14-19 cells to generate 11 and 32 MPA/X-resistant (MPA/X^r^) colonies, respectively. No MPA/X^r ^colonies developed in the control transfections lacking pMC-Cre. Of the 11 Rht14-10-derived colonies, 9 had lost GFP expression and became sensitive to MPA/X when tet was added (not shown). Of the 32 Rht14-19-derived colonies, 22 had lost GFP expression and became sensitive to MPA/X when tet was added (not shown). Nine GFP-negative, MPA/X^r ^clones from each parental clone were analysed by genomic PCR, and all were positive for the 629 bp PCR product diagnostic for the expected integration (Fig. 4A,B). Four insertion clones, one derived from Rht14-10 (10IN5) and three from Rht14-19 (19IN3, 19IN4 and 19IN5), were analysed by Southern blot and all showed the expected change in structure (Fig. 4A,C). Together, these results indicate that the desired insertion event occurred at frequencies of 1.8 × 10^-5 ^and 4.4 × 10^-5 ^per transfected cell in clones Rht14-10 or Rht14-19, respectively (Table 1).

### Flp-mediated deletion

**Figure 5:**
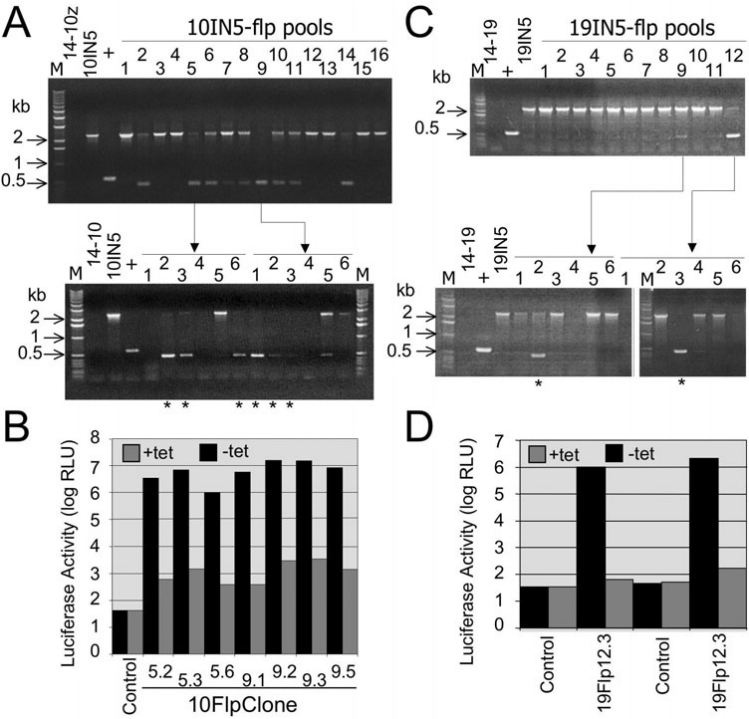
**Flp-mediated deletion and resulting luciferase expression in Rht14-10IN5 and Rht14-19IN5**. **A**. Ethidium bromide stained agarose gel of electrophoresed PCR products generated with the primers O2 and O4 (Fig. 4A) and cell pellets of Rht14-10, Rht14-10IN5, pTIGHTluc (+) and Rht10IN5flp pools (top, 1–16) or clones constituting Rht10IN5flp pools 5 and 9 (bottom). Positive controls (+) used pTIGHTluc DNA (expected product: 517 bp). **B**. Luciferase activity in lysates from clones indicated (*) in A, or control cells (Rh14-10), grown in the absence or the presence (for 48 h) of tet. **C**. Ethidium bromide stained agarose gel of electrophoresed PCR products generated with the primers O2 and O4 (Fig. 4A) and cell pellets of Rht14-19, Rht14-19IN5, and Rht19IN5flp pools (top, 1–12) or constituent clones of pools 9 and 12 (bottom, 1–6). Positive controls (+) were as in A. **D**. Luciferase activity in lysates from clones indicated (*) in C, or control cells (Rht14-19), grown in the absence or the presence (for 48 h) of tet. Results of two experiments are shown.

Two insertion clones (10IN5 and 19IN5), one of each parental type, were chosen for Flp-mediated deletion. Cells grown with tet (without MPA/X) for at least 72 h were lipofected (without [10IN5] or with [19IN5] tet for 4 h; see methods) with the Flp expression plasmid pCAGGS-flpe (Cambion), and cultured at low density with tet until colonies appeared. Colonies were analysed by genomic PCR. For Flp-treated 10IN5 cells, 16 pools (6 clones/pool) were assayed and 9 pools produced the predicted 472 bp PCR product (Fig. 4Ac) indicative of Flp-mediated deletion (Fig. 5A). Individual clones from two positive pools (5 and 9) were analysed by the same PCR assay and 6/12 were positive for the 472 bp product (Fig. 5A). The efficiency of flp-mediated deletion was thus at least 0.14 (13/96), and probably closer to 0.28 (6/12 × 9/16), events per transfected cell. A Southern blot of two such clones (10flp9.2 and 10flp9.3) was consistent with the predicted structures (Fig. 4A,C). The 7 Flp-deleted clones showed highly stringent tet-regulated luciferase expression (Fig. 5B). Above-background levels of uninduced luciferase expression were detectable, but all clones showed induction ratios approaching 10,000-fold, consistent with the GFP inducibility of parental clone Rht14-10.

For Flp-treated 19IN5 cells, 12 pools (6 clones/pool) were assayed by PCR (Fig. 5C). Two pools (9 and 12) were positive, each due to the presence of a single clone 19flp9.2 and 19flp12.3, respectively. The relatively low efficiency of Flp-mediated deletion in 19IN5 (0.03 [2/72] events per transfected cell) probably reflects a lower efficiency of pCAGGS-flpe delivery due to the inclusion of tet in lipofection mix of that particular transfection. Southern blot (Fig. 4C) and PCR analysis (Fig. 5C), showed that 19flp9.2 was impure, the majority of cells being of parental (19IN5) type. Southern analysis of the other flp-deleted clone (19flp12.3), however, confirmed that it had undergone the expected deletion (Fig. 4C). Clone 19flp12.3 showed highly stringent tet-regulation of luciferase expression with an induction ratio of more than 10,000-fold and uninduced expression levels barely above background (Fig. 5D).

### Use of ScIn-2 to generate cells with inducible I-SceI or Rad52 expression

**Figure 6:**
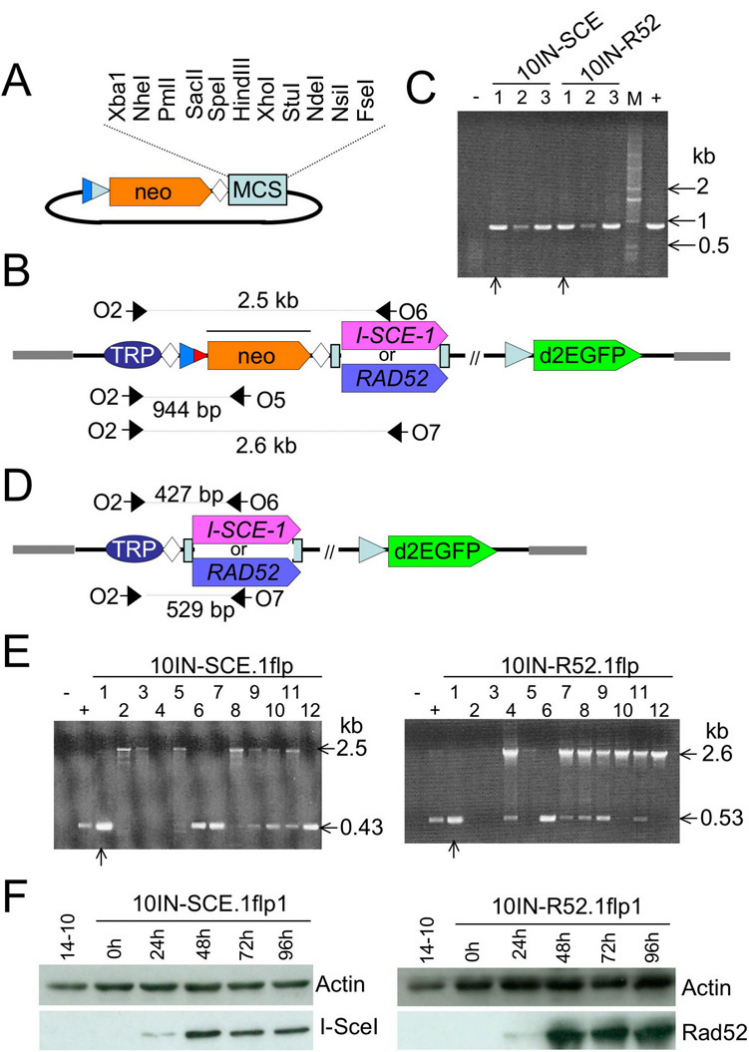
**Use of ScIn-2 method for stringent tet-regulated expression of I-*Sce*I and Rad52**. **A**. Schematic (not to scale) of pIN2-neoMCS. DNA is represented as in Fig 1. MCS, multiple cloning site. **B**. Schematic (not to scale) of target locus on Rht14-10 after Cre-mediated insertion of pIN2-neoSCE or pIN2-neoR52. **C**. Ethidium bromide stained agarose gel of electrophoresed PCR products generated with the primers O2 and O5 on cell pellets of three pIN2-neoSCE- and three pIN2-neoR52-transfected Rht14-10 derivatives. Negative (-) and positive (+) controls were, respectively, no cells and cells (TetNeo, see methods) with an integrated tet-regulated neo cassette (expected product: 944 bp). Clones chosen for flp-mediated deletion (10IN-SCE.1 and 10IN-R52.1) are arrowed. **D**. As in B but after Flp mediated deletion. **E**. Ethidium bromide stained agarose gel of electrophoresed PCR products generated with the primers O2 and O6 on clones derived from 10IN-SCE.1 (1–12, left) and primers O2 and O7 on clones derived from 10IN-R52.1 (1–12, right). Control reaction contained no cells (-) or genomic DNA generated from pools of flp-treated pIN2-neoSCE/pIN2-neoR52 cells (+). Clones chosen for further analysis (10IN-SCE.1flp1 and 10IN-R52.1flp1) are arrowed. **F**. Immunoblot analysis of I-*Sce*I and Rad52 expression in Rht14-10 and, respectively in 10IN-SCE.1flp1 or 10IN-R52.1flp1 at the indicated times after removal or tetracycline.

Having validated the ScIn method, we put it into practice using ScIn-2 with genes for I-*Sce*I or Rad52 as our GOIs. For these experiments a modified insertion pIN-2 vector (pIN2-neoMCS) was used with a different selectable marker gene (*neo *in place of *gpt*) followed by a multiple cloning site (Fig. 6A). The open reading frames (ORFs) encoding I-*Sce*I or Rad52, the former with a N-terminal Haemaglutinin (HA) tag, were cloned into pIN2-neoMCS to generate pIN2-neoSCE and pIN2-neoR52. These plasmids were co-lipofected with pMC-Cre into Rht14-10 and G418^r ^colonies were selected, > 96% of which had lost GFP expression. Of six such clones tested, three each of the pIN-neoSCE- and pIN-neoR52-transfected clones, all were found to become sensitive to G418 in tet and to be positive in PCR assays for targeted integration (Fig. 6B,C). In this way site-specific insertion frequencies of between 3.3 and 4.3 × 10^-4 ^were estimated for pIN2-neoMCS and it derivatives, at least 20-fold higher than with pIN-2.

Two clones, 10IN-SCE.1, 10IN-R52.1 (with pIN2-neoSCE and pIN2-neoRAD52 integrated, respectively) were grown in +tet medium, lipofected (- tet for 4 h; see methods) with pCAGGS-flpe and plated at low dilution in +tet medium. The majority of the resulting colonies were positive in a PCR assay for the deletion event, suggesting highly efficient Flp-mediate deletion (Fig. 6C–E). Two clones of high purity (10IN-SCE.1flp1, 10IN-R52.1flp1) as judged by PCR were analysed further. Immunoblot analyses confirmed the expected tet-regulated expression of the appropriate GOI (I-*Sce*I or Rad52) in these clones (Fig. 6F). Expression in the presence of tet was undetectable indicating, as far as is possible by immunoblotting, highly stringent tet-regulation.

To test for functionality of the induced I-*Sce*I, and as a more robust test for the stringency of tet-regulation, 10IN-SCE.1flp1 was transfected with pDRneo [31]. This substrate for homologous recombination carries a functional hygromycin resistance cassette flanked by two defective *neo *cassettes, one being disrupted in its coding sequence by the 18 bp recognition site for I-*Sce*I. Cleavage of the first *neo *cassette by I-*Sce*I greatly stimulates its conversion to a functional *neo *cassette by homology directed DNA repair using the second *neo *cassette as a template. Before transfection with pDRneo, the purity of 10IN-SCE.1flp1 was further demonstrated by plating three million cells in G418 without tet: no colonies formed even though parental 10IN-SCE.1 cells grow in these conditions. Following transfection of 10IN-SCE.1flp1 with pDRneo, three Hygro^r ^clones, selected and expanded with tet in the growth medium, were tested for the presence of G418^r ^cells after a period (2 days) of growth with or without tet. The results (Table 2) show that I-*Sce*I induction causes > 10^4^-fold stimulations in gene conversion, with absolute frequencies reaching 3–7% of all cells. These effects are at least as pronounced as those previously described after transient transfection of an I-*Sce*I expression construct [31]. Variations between clones in the absolute frequencies of G418^r ^are most likely caused by different chromosomal position effects on the integrated pDRneo. The low frequencies of G418^r ^colonies (1–5 × 10^-6^) detected in the continuous presence of tet are likely to reflect spontaneous recombination of the pDRneo substrate rather than recombination stimulated by residual I-*Sce*I expression: similar frequencies of spontaneous G418^r ^have been described for pDRneo in HT1080 [32], HEK293 [32] and CHO [31] cells that have never been transfected with an I-*Sce*I gene. Furthermore, these frequencies are no higher than the spontaneous G418^r ^frequency (1.3 × 10^-5^) observed for a hygro^r ^clone derived from pDRneo-transfected Rht14 cells (Table 2). This frequency was also unaffected by tet removal, excluding the possibility that tet reduced G418^r ^colony formation independently of its effect on I-*Sce*I expression. Taken together, these results clearly demonstrate that I-*Sce*I is functional and regulated with very high stringency following its targeted integration by use of the ScIn-2 method.

## Discussion

The "Screen and Insert" (ScIn) methods described here provide convenient and reproducible ways to isolate clones in which transgene expression is stringently regulated by tet. A great variety of modifications and improvements to the original Tet-Off system have been reported. Versatility, convenience, stringency and stability have been variously extended by the development of the reverse tTA (Tet-On) system [33], the combined use of tet-regulated activation and repression [34,35], systems for single-vector delivery and/or autoregulation [36-44] and by modifications to the TRE [45-48] or the transactivator protein/gene [18,28,49-52]. Despite such improvements, the tet system remains subject to the influence of chromosome position effects, complicating the isolation of optimally regulated clones. The ScIn methods address this problem.

The key to ScIn is the combination of a powerful GFP-based screen to identify optimal chromosomal loci, and the use of SSR to target the insertion of transgenes at such sites. Screening by flow cytometry is valuable because it allows lines with even very small amounts of leaky expression, or with cellular heterogeneity of expression, to be eliminated. Furthermore, for a given cell line, one can envisage collecting a range of clones with different induction characteristics, from which one can choose the clone most suitable for a particular application. The ease with which many clones can be screened increases the chances of finding one in which optimal regulation is achieved. Although we screened clones individually here, it may be even more convenient in future to use flow sorting to purify and clone the best cells from a population of transfectants.

It is important to note that, once screening has been used to identify a clone (e.g. Rht14-10) with a stringently regulated reporter, the use of SSR to insert the GOI at the reporter locus is relatively labour-unintensive and can be used repeatedly for different GOIs. In ScIn2, for example, the following three steps are now sufficient to generate an HT1080 clone with any GOI under highly stringent tet-regulation: i) Clone GOI into multiple cloning site of pIN2-neoMCS and co-transfect with pMC-Cre into Rht14-10, selecting for G418^r ^colonies. ii) Expand a G418^r^/GFP^- ^colony, confirm G418^r ^is tet-sensitive (optional), transfect with pCAAGflpe (or similar) and plate at low density in tet. iii) Identify one of the >10% of colonies that is no longer G418^r ^when tet is removed.

ScIn has at least three advantages over the standard approach of immunoblot-screening of multiple clones with randomly integrated GOI. First, no screening for GOI expression is required to identify the desired clone. Second, tet-regulation of the GOI is likely to be much more stringent and lacking in cellular heterogeneity. Third, the same protocol can be used repeatedly with different GOIs to generate clones whose phenotypes can be compared without the complication of differential expression levels. These advantages will be attractive for a range of studies, including in both gain- and loss-of-function experiments. The expression of I-*Sce*I provides a good illustration of the former. Leaky expression of this endonuclease will lead to repeated cutting and repair of any I-*Sce*I recognition sites in the host genome, until inaccurate repair by non-homologous end-joining [53] results in the loss of I-*Sce*I sites. The ability to induce DSB, and measure cellular responses, will therefore be irreversibly lost if expression is leaky. The importance of minimal leakiness in loss-of-function experiments is illustrated by systems in which a tet-regulated transgene is expressed in cells whose corresponding endogenous alleles have been inactivated [13,14]. Switching off the tet-regulated transgene in such systems will produce a true null phenotype only when there is no leakiness.

Success of the ScIn method depends on optimal reporter gene regulation from the target construct and this was achieved best with pTARG4. Stringent regulation from the other contructs (pTARG1-3) was limited by use of the original TRE and/or by host cells that expressed tTA rather itTA. Although we saw no signs of structural instability or unstable gene expression at pTARG4 loci, the persistence of prokaryotic vector sequences at transgene loci can in some circumstances be problematic [54-56]. Removal of unwanted vector DNA from target and insertion constructs prior to transfection might therefore be considered desirable. For target constructs this may be achieved simply by digestion with appropriate restriction enzymes. For insertion constructs, which must remain circular, this might be achievable by inclusion of a second *loxP *site and use of Cre recombinase *in vitro*.

The use of SSR to integrate transgenes at sites that support tissue-specific or ubiquitous expression in mice is established [57,58]. For tet-regulated expression, SSR has been used to target either to defined loci, such as the *HPRT *[59] or *ColA1 *[19] genes in mouse embryonic stem (ES) cells, and the *DHFR *locus in Chinese Hamster Ovary (CHO) cells locus[22], or to specific but undefined loci in e.g. CHO [22], human cervical [22] and rat pancreatic cells [20]. While these systems introduce the valuable aspect of reproducibility into transgene integration in a given cell line, the chosen target sites are not necessarily optimal for stringency or for cellular homogeneity of induction. Furthermore, establishing these systems in new cell lines is demanding.

In an approach more closely related to ScIn, Puttini *et al *[60] used I-*Sce*I-stimulated homologous recombination (HR) to target transgenes to a locus that was identified by its ability to support stringent tet-regulation of another reporter, secreted alkaline phosphatase (SEAP). In practice, this approach was limited by the low efficiency of HR-mediated integration, the inability to use SEAP assays to assess cellular heterogeneity and the presence of a promoter/enhancer close to the TRE. In another approach related to ScIn, a tet-regulated *gpt *gene was linked to a promoterless GOI and randomly integrated into the genome [27]. After selecting clones with optimally-regulated gpt, SSR was use to invert the DNA segment carrying *gpt *and GOI, placing the GOI under control of a TRE. This system suffered from the low stringency of gpt selection and the need to select optimally-regulated clones for each new GOI.

Emphasis in this paper has been on the use of transgenes for the analysis of gene function in cultured cells. The ScIn may also be valuable when extended to mice. For this, however, methods must be developed to screen for ES cell clones that support tet-regulated GFP expression not just in culture, but also in all tissues of ES-derived animals. Provided its tTA/rtTA gene can be deleted (e.g. by SSR), the chosen clone can then be used repeatedly for the targeted insertion of any GOI, and the generation of mice to be crossed with transgenic lines chosen for their tissue-specific expression of tTA/rtTA.

## Conclusion

The screen and insert approach described is a highly effective way to achieve stringent and reproducible tet-regulated transgene expression. Reagents developed here (e.g. clone Rht14-10 and the insertion vector pIN2-neoMCS) can be used immediately in a simple 3-step protocol to achieve such expression in an HT1080 background. Furthermore, additional reagents we describe (e.g. pTARG4), can be used to establish the select and insert method in other cell lines. The method is particularly attractive for analyses of gene function in situations where the gene product is likely to have biological effects even when expressed at very low levels and/or where the effects of several related genes are to be compared. Cell lines expressing stringently regulated I-*Sce*I or Rad52, generated in the course of this study, will be useful in studies of DNA damage and repair.

## Methods

### Target plasmids

#### pTARG1

A lox71 site (annealed oligonucleotides 5'-TACCGTTCGTATAGCATACATTATACGAAGTTAT-3' and 5'-CTAGATAACTTCGTATAATGTATCGTATACGAACGGTAGC-3') was ligated to the *Sac*II and *Xba*I sites of pUHD10-3 ([29], kindly donated by H. Bujard, ZMBH, Heidelberg) to create pTRElox71. To generate pTARG1, an *Xba*I fragment from pEGFP (Clontech), carrying the EGFP open reading frame (ORF), was inserted at *Xba*I site downstream of the *lox71 *site of pTRElox71.

#### pTARG2

The ORF of *d2EGFP *(from pd2EGFP, Clontech) was isolated as a *Not*I/*Age*I fragment and inserted into the *Not*I/*Age*I sites of pTRElox71.

#### pTARG3

A lox71 site (annealed oligonucleotides 5'-AATTCTACCGTTCGTATAGCATACATTATACGAAGTTATACTAGTG-3' and 5'-GATCCATCAGTATAACTTCGTATAATGTATGCTATACGAACGGTAG3') was cloned into the *EcoR*I and *Bam*HI sites of pTRE-TIGHT (Clontech), creating pTlox71. To generate pTARG3, the ORF of *d2EGFP *(from pd2EGFP, Clontech) was isolated as a *Not*I/*Bam*HI fragment and inserted into the *Not*I and *Bam*HI sites of pTlox71.

#### pTARG4

An FRT site (annealed oligonucleotides 5'-AATTAGATCTGAAGTTCCTATTCTCTAGAAAGTATAGGAACTTC-3' and 5'-AATTGAAGTTCCTATACTTTCTAGAGAATAGGAACTTCAGATCT-3') was cloned into the *Hind*III/*Nco*I sites of pTARG3.

### Insertion plasmids

#### pIN-1

A *lox66 *site (annealed oligonucleotides 5'-TCGAGAATTCATAACTTCGTATAGCATACATTATACGAACGGTAG-3' and 5'-AGCTCTACCGTTCGTATAATGTATCGTATACGAAGTTATGAATTC-3') was cloned into the *Xho*I/*Hind*III sites of pGL3-Basic (Clontech) to generate plox66luc. To generate pIN-1, pIRESHyg3 (Clontech) was digested with *Xho*I/*Nhe*I and the IRES-Hyg fragment was cloned into the *Xba*I/*Sal*I site of plox66luc, downstream of the luciferase ORF.

#### pIN-2

The *gpt *ORF was removed from pBSgpt [61] as a *Bam*HI/*Bgl*II fragment and cloned into the *Bgl*II site of pGL3-Basic (Clontech) to make pGL3gptluc. A *lox66 *site (annealed oligonucleotides 5'-CTAGATAACTTCGTATAGCATACATTATACGAACGGTAGAAT-3' and 5'-TCGAATTCTACCGTTCGTATAATGTATGCTATACGAAGTTAT-3') was then cloned into the *Nhe*I/*Xho*I sites of pGL3gptluc upstream of the luciferase ORF to make plox66gptluc. To generate pIN-2, an FRT site (annealed oligonucleotides 5'-AGCTGAAGTTCCTATTCTCTAGAAAGTATAGGAACTTCGAATT-3' and 5'-CATGAATTCGAAGTTCCTATACTTTCTAGAGAATAGGAACTTC-3') was cloned into the *Hind*III/*Nco*I sites of plox66gptluc located between the luciferase and *gpt *ORFs.

#### pINneoMCS

An MCS (annealed oligonucleotides 5'-CGCTAGCAGCTGGTCCGCGGACTAGTCCCGGGAAGCTTCTCGAGAGGCCTCATATGCATGCCATGGCCGG-3' and 5'-CCATGGCATGCATATGAGGCCTCTCGAGAAGCTTCCCGGGACTAGTCCGCGGACCAGCTGCTAG-3') was inserted into *Bst*BI/*Fse*I sites of pIN-2, removing the luciferase ORF, to create pIN2-MCS. pSV2neo (Southern *et al*., 1982) and plox66gptLuc were cut with *Bgl*II and *Bam*HI and neomycin ORF was cloned downstream of the *lox*66 site to create plox66Neo. To create pIN2-neoMCS, pIN2-MCS and plox66neo were cut with *Bgl*II and *Pfm*I and the *gpt *ORF was replaced with the neomycin ORF.

#### pIN2-neoSCE and pIN2-neoR52

pIN2-neoMCS was cut with *Eco*RI, end-filled then cut with *Sal*I. pFB580 (human RAD52 cDNA cloned into pUC18, a kind gift from F. Benson and S. West, Cancer Research UK, London) and pCMV3xnls-I-*Sce*I ([62], a kind gift from M. Jasin, Sloane Kettering Institute, New York) were also cut with *Eco*RI, end-filled then cut with *Sal*I, to release the *RAD52 *and I-*Sce*I ORFs, respectively. *RAD52 *and *I-SceI *ORFs were then cloned into pIN2-neoMCS to generate pIN2-neoR52 and pIN2-neoSCE, respectively.

#### plox66Neo

plox66Neo (see above) and pSV2neo were digested with *Bgl*II and *Bam*H1 and the neo ORF was cloned downstream of the lox66 site.

### Other plasmids

A plasmid (pRK5-itTA) encoding the improved transactivator itTA [28] was kindly donated by R. Sprengel, Max-Planck-Institute, Heidelberg. The itTA ORF was cloned as an *Eco*R1/*Bcl*I fragment into the *Eco*RI/*Bcl*I sites of pZeoSV (Invitrogen), to create pZeoSVitTA. pTIGHTgpt was made by inserting the *gpt *ORF as a *Bgl*II/*Bam*H1 fragment from pBSgpt into the *Bam*H1 site of pTRE-TIGHT.

### Cell culture

Conditions used for the culture of HT1080 cells and derivatives have been described previously [63]. When required the medium was supplemented with one or more of the following drugs: 5-azacytidine (1 μM), hygromycin B (100 μg/ml), mycophenolic acid (10 μg/ml), puromycin (0.4 μg/ml), tetracycline (1 μg/ml), xanthine (100 μg/ml) and zeocin (200 μg/ml). HT-2 cells have been described [27] and were also called HTET [13]. A telomerase-immortalised human retinal epithelial cell line (hTERT-RPE1; Clontech) was cultured in a similar manner to HT1080 cells.

### Cell lines generated by electroporation

Electroporation with a Gene Pulser (BioRad) was as described [64]. Rht14 cells were made by electroporation of HPRT^+ ^HT1080 cells with 10 μg *Bgl*II-linearised pZeoSVitTA. Zeocin^r ^colonies were screened by lipofection (see below) with pTIGHTLuc (Clontech) grown in the presence or absence of tetracycline. After 48 hours, lysates were prepared and assayed for luciferase. Rht14 was chosen from among 24 colonies for its low luciferase activity in the presence of tet and its high induction ratio. Co-electroporation was used to generate clones with randomly integrated target plasmids. Target plasmid (20 μg) was linearised with *Pvu*I (pTARG1) or *Bgl*I and *Ssp*I (pTARG2, pTARG3, pTARG4), gel-extracted, ethanol-precipitated and co-electroporated with 1 μg of similarly purified, *Spe*I-linearised pBL-PuroR [32], into Rht14 cells. Puro^r ^colonies identified as GFP-positive by fluorescence microscopy (Zeiss Axiovert S100TV microscope) were picked for further analysis.

### Lipofection

Lipofectamine 2000 (Invitrogen) was used for delivery of insertion constructs and transient expression of Cre and Flp recombinases and luciferase. 24 hours prior to transfection, 250,000 cells were plated into 6-well plates in 2 ml of antibiotic-free (- tet, unless otherwise stated) medium. On the day of transfection a DNA/Lipofectamine/OptiMEM mix was prepared (0.5 ml/well) according to the manufacturer's instructions and incubated with the cells for 4 hours, after which the mix was replaced with fresh medium. For Cre-mediated insertion, 2 μg of insertion construct and 2 μg of pMC-Cre15 (kindly donated by H. Gu, University of Köln) were used per well. The following day, the cells were seeded into 9 cm plates (10^5 ^cells per plate) and the appropriate drug selection was added after a further 24 hours. For *flp*-mediated excision, 4 μg of pCAGGS-flpe (Cambion) was used per well and cells were plated the following day at low density (50–500 cells per 9 cm [diam] dish) in medium with tet. To analyse the effect of AZC on luciferase expression from pTIGHT-luc, clone T15 cells were grown with or without AZC for 24 h, then plated in antibiotic-free medium and lipofected 24 h later (as above) with pTIGHT-luc (4 μg). After a further 24 h lysates were prepared and assayed for luciferase (see below).

### Flow cytometry

Cells were grown in 6-well plates with or without tet for 48–72 hours (except where longer times are indicated) prior to FACS analysis. Cells, generally no more than 80% confluent on the day of analysis, were trypsinised and resuspended in cold phosphate buffered saline (PBS) at a density of 1000 cells per μl. 40,000 cells were analysed in a FACScan machine (Becton Dickinson) with an argon laser tuned to 488 nm (FL-1; with fluorescence channel FL-3 as a control). Acquisition, storage, and analysis of data were carried out using CellQuest software (Becton Dickinson).

### Immunoblots

Immunoblots were carried out as described previously for Rad52 [65]. For GFP detection, a primary monoclonal antibody was used (Clontech, 632380; 1/1000 dilution) and a secondary, horseradish peroxidase-conjugated goat anti-mouse immunoglobulin antibody (Sigma, P-0447; 1/1000 dilution) was used. For HA-tagged I-*Sce*I detection, the primary antibody was a rat monoclonal (Roche, 3F10; 1/500 dilution) and the secondary a horseradish peroxidase-conjugated goat anti-rat immunoglobulin antibody (Sigma, A-9037; 1/1000 dilution)

### Polymerase Chain Reactions (PCR)

Cell pellets (100–10,000 cells) were resuspended in a 25 μl Taq polymerase buffer (Qiagen) containing pronase (0.6 μg/μl), incubated at 50°C for one hour, 95°C for 10 minutes and then placed on ice. A mix (25 μl) containing nucleotides (Pharmacia, 0.5 μM), Taq polymerase (Qiagen, 1.25 U) and oligonucleotides (100 ng each) dissolved in in Taq polymerase buffer was added. Annealing temperatures (T_a_) for the various primer pairs were 60°C (O2/O4 and O1/O3), 63°C (O2/O5), and 57°C (O2/O6 and O2/O7). Temperature sequences were: 95°C (10 min.) then 30 cycles of 95°C, T_a_, and 72°C (1 min each) then 72°C (10 min.). Primers were: O1 (5'-ACGAGGCCCTTTCGTCTTCA-3'), O2 (5'-TTTAGTGAACCGTCAGATCGCC-3'), O3 (5'-CCACGGCTTACGGCAATAATGC-3'), O4 (5'-CCCCTTTTTGGAAACGAACAC-3'), O5, (5'-AGAAGGCGATAGAAGGCGATGC-3'), O6 (5'-ACTCGAACTGCATACAGTAG-3') and O7 (5'-CCAAAGATAAAGCCTTGGAC-3').

As positive controls for the DNA template, ~10 pg of the indicated plasmid DNA, or genomic DNA from 1000 pelleted cells of the indicated cell line, were used. TetNeo cells have a tet-regulated *neo *gene and are described in detail elsewhere [30]. Briefly, plox66Neo was inserted by Cre-mediated recombination into tTERT-RPE1 cells with an integrated pTARG4 supporting tightly regulated GFP. PCR products were analysed by standard agarose gel electrophoresis with 1 kb ladder marker DNA (Invitrogen).

### Southern blots

Standard methods were used, as previously described [61]. The GFP probe was a 727 bp *Nco*I fragment from pd2EGFP [Clontech]). Marker DNA was end-labelled 1 kb ladder (Invitrogen).

### Luciferase assays

A commercial kit (Promega) was used according to manufacturers instructions. Briefly, cells were seeded at 10^5 ^cells per 3.5 cm (diam.) well, incubated in medium with or without tet for 48 hours, then washed with PBS and agitated gently for at least 20 minutes with 500 μl passive lysis buffer. The cell lysate was cleared by centrifugation and 20 μl added to 100 μl of luciferase assay reagent in a luminometer tube. The relative light units (RLU) were measured immediately in a Biorbit 1253 luminometer. The average of ten RLU measurements taken at 2-second intervals was recorded.

### Recombination of pDRneo

To estimate the recombination frequency when I-*Sce*I was not expressed, 3 million cells were distributed in three 15 cm (diam.) plates and selected in G418 and tetracycline and colonies counted after 14 d. To determine the frequency after I-*Sce*I induction, cells were grown in the absence of tet for 48 h and then plated (at 50, 100, 250 cells per 9 cm [diam.] plate) in G418 and tetracycline and colonies counted after 14 d. To account for the plating efficiency, the proportion of G418^r ^clones was calculated as a fraction of the total number of colonies formed when compared to the amount generated on equivalent unselected (no G418) plates. Rht14/DRneo cells were analysed in the same way except that tet was either present or absent from the growth medium throughout, and for 48 h prior to, G418 selection.

## Authors' contributions

RB carried out most of the experimental design and implementation, and prepared the draft manuscript. AMP carried out some preliminary studies including the generation of clone 6 (Fig. 2). ACGP conceived the basic approaches taken, carried out some experimental work, supervised the project, and prepared the final manuscript. All authors read and approved the final manuscript.

## Figures and Tables

**Table 1:** Frequencies of Cre-mediated insertion events

^a ^Host cell	Insertion construct	^b ^Cre	Drug Selection	^c ^Colonies	^d ^GFP-neg	^e ^Tet	^f ^PCR/SB	^g ^I
Rht14-19	pIN-1	+	hyg	53	47/48	47/48	10/10	10
Rht14-19	pIN-1	-	hyg	0	-	-	-	0
Rht14-10	pIN-2	+	MPA/X	11	9/11	9/11	9/9	1.8
Rht14-10	pIN-2	-	MPA/X	0	-	-	-	0
Rht14-19	pIN-2	+	MPA/X	32	22/33	22/33	9/9	4.4
Rht14-19	pIN-2	-	MPA/X	0	-	-	-	0
Rht14-10	pIN2-neoMCS	+	G418	188	181/188	NT	NT	36
Rht14-10	pIN2-neoSCE	+	G418	169	163/169	3/3	3/3	33
Rht14-10	pIN2-neoR52	+	G418	226	217/226	3/3	3/3	43

^a ^Each row represents data for the transfection of 5 × 10^5 ^cells.

^b ^Indicates whether pMC-Cre was (+) or was not (-) co-transfected.

^c ^Total number of drug-resistant colonies generated.

^d ^Colonies that did not express GFP.

^e ^GFP-negative colonies that became drug-sensitive on addition of tet.

^f ^GFP-neg/Tet^s ^colonies with expected structure detected by PCR or Southern Blot.

^g ^Cre-mediated insertion frequency expressed as events per transfected cell × 10^5 ^(assuming all GFP-neg clones are correctly inserted).

**Table 2:** Frequencies of G418^r ^colony formation in pDRneo-transfected cells

Cells	Frequency of G418^r^		Fold Induction
	+ tet	- tet	
^a ^10IN-SCE.1flp1/DRneo, Clone 1	4.7 × 10^-6^	7.2 × 10^-2^	1.5 × 10^4^
^a ^10IN-SCE.1flp1/DRneo, Clone 2	1.3 × 10^-6^	2.9 × 10^-2^	2.2 × 10^4^
^a ^10IN-SCE.1flp1/DRneo, Clone 3	1.0 × 10^-6^	3.5 × 10^-2^	3.5 × 10^4^
^b ^Rht14/DRneo	1.3 × 10^-5^	1.4 × 10^-5^	1.1

^a ^Three independent pDRneo-transfected 10IN-SCE.1flp1 clones were grown continuously with tet in the medium (+tet) or tet was withdrawn for 48 h. (-tet) prior to G418 selection (methods).

^b ^A single hygro^r ^clone, isolated after the transfection of Rht14 cells with pDRneo, was grown for 48 h. and then selected in G418, with tet either present (+tet) or not present (-tet) in the medium throughout (methods).
